# Kunitz Proteinase Inhibitors Limit Water Stress Responses in White Clover (*Trifolium repens* L.) Plants

**DOI:** 10.3389/fpls.2017.01683

**Published:** 2017-10-04

**Authors:** Afsana Islam, Susanna Leung, Aluh Nikmatullah, Paul P. Dijkwel, Michael T. McManus

**Affiliations:** Institute of Fundamental Sciences, Massey University, Palmerston North, New Zealand

**Keywords:** water deficiency, Kunitz Proteinase Inhibitor, *Trifolium repens*, white clover, proline accumulation

## Abstract

The response of plants to water deficiency or drought is a complex process, the perception of which is triggered at the molecular level before any visible morphological responses are detected. It was found that different groups of plant proteinase inhibitors (PIs) are induced and play an active role during abiotic stress conditions such as drought. Our previous work with the white clover (*Trifolium repens* L.) *Kunitz Proteinase Inhibitor* (*Tr-KPI*) gene family showed that *Tr-KPIs* are differentially regulated to ontogenetic and biotic stress associated cues and that, at least some members of this gene family may be required to maintain cellular homeostasis. Altered cellular homeostasis may also affect abiotic stress responses and therefore, we aimed to understand if distinct *Tr-PKI* members function during drought stress. First, the expression level of three *Tr-KPI* genes, *Tr-KPI1*, *Tr-KPI2*, and *Tr-KPI5*, was measured in two cultivars and one white clover ecotype with differing capacity to tolerate drought. The expression of *Tr-KPI1* and *Tr-KPI5* increased in response to water deficiency and this was exaggerated when the plants were treated with a previous period of water deficiency. In contrast, proline accumulation and increased expression of *Tr-NCED1*, a gene encoding a protein involved in ABA biosynthesis, was delayed in plants that experienced a previous drought period. RNAi knock-down of *Tr-KPI1* and *Tr-KPI5* resulted in increased proline accumulation in leaf tissue of plants grown under both well-watered and water-deficit conditions. In addition, increased expression of genes involved in ethylene biosynthesis was found. The data suggests that *Tr-KPIs*, particularly *Tr-KPI5*, have an explicit function during water limitation. The results also imply that the Tr-KPI family has different *in planta* proteinase targets and that the functions of this protein family are not solely restricted to one of storage proteins or in response to biotic stress.

## Introduction

Plant Kunitz proteinase inhibitors (KPIs) belong to the serine proteinase inhibitor (PI) group and numerous KPIs have now been identified from different plant species (Rawlings et al., 2016). In most plant species, the KPIs comprise a multi-gene family: there are 13 members in soybean (Jofuku and Goldberg, 1989), 21 in potato (Heibges et al., 2003a,b), 31 in poplar (Philippe et al., 2009) and more than 11 in white clover (Islam et al., 2015a). The gene family generally encodes proteins of approximately 18–22 KDa in size with a single reactive site (Laskowski and Kato, 1980). KPIs were originally proposed by Pusztai (1972) to function as storage proteins and this was later supported by other studies based on their occurrence in storage tissues like seed and tuber (Shewry et al., 1995; Shee and Sharma, 2008; Jørgensen et al., 2011). However, KPIs have been found to inhibit a range of proteinases like trypsin, chymotrypsin, elastase, subtilisin, cathepsin D and papain (Ritonja et al., 1990; Terada et al., 1994; Valueva et al., 2000; Heibges et al., 2003b; Major and Constabel, 2008) suggesting that, as a group, they have diverse proteinase targets. Indeed, the extensive range of *in vitro* inhibitory activities against different proteinases implies that KPIs have a wide range of *in planta* targets and functions. It was also suggested that KPIs act as a regulator of proteinases during germination and in defense-response during insect herbivory (Richardson et al., 1986; Xavier-Filho and Campos, 1989; Norton, 1991; Bauw et al., 2006; Hernández-Nistal et al., 2009). The activity of PIs including KPIs against insect digestive proteinases has led to the promotion of their use as tolerance determinants in transgenic plants (Green and Ryan, 1972; Hilder et al., 1987; Ryan, 1990; Jongsma and Bolter, 1997; Lee et al., 1999; McManus et al., 2005; Lima et al., 2011). Recent evidence also suggests that KPIs in plants are involved in programmed cell death, growth, and development (Karrer et al., 1998; Yeu et al., 2007; Kim et al., 2009; Laluk and Mengiste, 2011; Pereira et al., 2011; Boex-Fontvieille et al., 2015; Islam et al., 2015a). Moreover, changes in expression profile of *KPIs* in response to water limitation suggests a function for these proteins in abiotic stress, possibly by targeting specific proteinases and as such limiting their proteolytic activities (Downing et al., 1992; Kang et al., 2001; Desclos et al., 2008; Kidric et al., 2014).

To cope with water limitation, plants have evolved adaptive features and complex cellular signaling mechanisms to sense, respond, and survive (Shao et al., 2006; Bruce et al., 2007; Wu et al., 2007; Nakashima et al., 2009; Arbona et al., 2010; Harb et al., 2010; De Ollas et al., 2013). Although during stress phytohormone signaling pathways coordinate and integrate the whole plant response (Kreps et al., 2002; Bruce et al., 2007; Tardif et al., 2007; Wu et al., 2007), abscisic acid (ABA) plays a major role during drought (Fujita et al., 2011; Nakashima et al., 2014; Muñoz-Espinoza et al., 2015). ABA has been implicated in the early perception of water deficiency leading to the activation of stress-responsive genes and stimulation of stomatal closure to reduce water loss (Bray, 2002; Shinozaki et al., 2003; Xiong and Zhu, 2003; Verslues and Bray, 2006; Planchet et al., 2011). In addition, the involvement of ethylene (Et) has also been reported in drought-induced abscission as a mechanism to minimize water loss (Oh et al., 1997; Nikmatullah, 2009; Arraes et al., 2015). Recent studies also revealed that during abiotic stress, including water deficiency, ABA and Et act antagonistically where ABA limits Et production and associated inhibition of root elongation (Beaudoin et al., 2000; Sharp, 2002; Rosado et al., 2006; Wilkinson et al., 2012; Arraes et al., 2015). Another response to water stress is the accumulation of proline, which has been reported to act as tolerance factor in many plant species (McManus et al., 2000; Verdoy et al., 2006; Mattioli et al., 2008; Szabados and Savouré, 2010; Planchet et al., 2014). Several studies have also reported the association of ABA and proline accumulation in the model plant species *Arabidopsis thaliana* and *Medicago truncatula* during water stress (Strizhov et al., 1997; Szabados and Savouré, 2010; Planchet et al., 2011, 2014). However, independent of ABA and Et, many transcription factors and defense associated genes are also induced by water stress perception and play a role in stress adaptation or tolerance (Nakashima et al., 2014).

Both proteinases and proteinase inhibitors have been reported to be induced by water limiting conditions (Richards et al., 1994; Snowden et al., 1995; Fan and Wu, 2005; Shinozaki and Yamaguchi-Shinozaki, 2007; Yamaguchi et al., 2010; Kidric et al., 2014), though the hormonal control of this induction is unknown. The induced cellular proteolysis in water limitation is a complex process and it is difficult to establish a link between a specific group of proteinase and their corresponding inhibitor families (Degenkolbe et al., 2009; Simova-Stoilova et al., 2010; Mosolov and Valueva, 2011; Kidric et al., 2014). Among different PI families, cysteine PIs (cystatins) are widely studied in relation to water limitation (Doip et al., 2004; Zhang et al., 2008; Munger et al., 2012; Jangpromma et al., 2014; Quain et al., 2014; Kunert et al., 2015), whereas relatively little is known about the function of KPIs during water limitation (Downing et al., 1992; Kang et al., 2001).

Previously we described four phylogenetically distinct members of the *KPI* gene family from white clover (*Trifolium repens* L.; *Tr-KPI*) and showed that a range of developmental and biotic stress-associated cues regulate their expression (Islam et al., 2015a,b). It was also shown that knock-down of these *Tr-KPIs* affected plant development, increased oxidative stress and revealed altered transcription of cellular signaling genes. The result suggested that for this gene family, a delicate regulation of *Tr-KPI* expression is required for the maintenance of a homeostasis critical for cell function. Therefore, we hypothesized that abiotic stress, such as water deficiency, also regulates the expression of *Tr-KPI* gene family members and that their role in maintaining cellular homeostasis affect the plants’ response to drought stress. In the current study, we have addressed the association of the *KPI* gene family as a stress-related factor during water limiting conditions in a non-model pasture plant species white clover. Using typical water deficiency and priming to a previous water deficiency, we showed that the expression of the members of *Tr-KPI* gene family were induced in distinct ways. The knock-down RNAi lines for the specific members of the gene family showed high proline accumulation in well-watered and water deficit condition with alteration of Et biosynthesis genes. The results suggest that the regulated expression of white clover *Tr-KPI* genes is important during water limitation.

## Materials and Methods

### Plant Materials and Growth Conditions

Two cultivars and one ecotype of white clover with different water requirements, namely, Kopu, Huia and Tienshan, were provided by AgResearch Grasslands, Palmerston North, New Zealand. The RNAi knock-down lines are described in Islam et al. (2015a) and are in the Huia background. The small-leaved white clover ecotype Tienshan is considered to display some drought tolerance; cv. Kopu with its large leaves is more drought susceptible; while cv. Huia with medium sized leaves is considered to be intermediate in terms of water requirement (Bosch et al., 1993; Hofmann et al., 2003; Hofmann and Jahufer, 2011). Plants arising from a single seed were selected randomly and maintained as stock plants. Clonal material for the experiments was taken from the stock plants as follows: the apical part of the stolon from the stock plants was excised just proximal to node four and all leaves were excised except the first emerged leaf. The stolon cuttings were then placed in pots containing vermiculite and were watered regularly with half strength Hoagland’s solution (Gibeaut et al., 1997) and allowed to develop roots for at least 1 week. Healthy and morphologically similar rooted stolons were then transferred to soil. A single stolon was grown in a 5L capacity pot for Tienshan and Kopu before transferring to environment-controlled rooms of the New Zealand Climate Environment Laboratory (NZCEL, Plant and Food Research, Palmerston North) as described by Nikmatullah (2009). The plants were grown for 1 week for acclimation before being subjected to water limiting conditions. For experiments with the ‘Huia’ cultivar and derived RNAi knock-down lines, a single four-noded stolon was grown in a 1:1 vermiculite:perlite mixture per pot to facilitate harvesting of roots for RNA extraction as it was difficult to remove attached soil particles from the root structure of soil-grown plants. The plants were grown in 1.2-L-capacity pots and were watered regularly with 0.5× Hoagland’s solution at a plant growth room at Massey University. All plants were grown in a controlled temperature room at 22°C during the day and 14°C during the night, with a relative humidity (RH) of ∼65% and a light intensity of 150 μE over a 14 h photoperiod. All the plants, both before and during the treatment, were grown in controlled temperature rooms, as mentioned. Two sets of plants for the treatments were maintained separately in the controlled temperature room. Further, pots were moved every other day to minimize environmental variation due to placement of pots. While harvesting the leaf and root materials, the plants were selected randomly to eliminate any biasness. The development of RNAi knock-down lines for the four *Tr-KPIs* namely *Tr-KPI1*, *Tr-KPI2*, *Tr-KPI4*, and *Tr-KPI5* is described in detail in Islam et al. (2015a). The positive RNAi lines (T_0_) and a number of non-expressing lines (designated as controls, C) were transferred to soil and maintained in a temperature-controlled containment glasshouse at 22°C, with ambient light and humidity. These were used as stock plants for the RNAi and control lines from which clonal stolon cuttings were excised, as described above.

### Water Limitation Experiments

To examine the involvement of *Tr-KPI* gene family members in drought stress, two different water with-holding regimes were applied to either produce plants that were newly exposed to a water deficit or those that had first been pre-exposed (pre-conditioned) to a water deficit period: (i) a Non-PreStress (NPS) treatment consisting of a direct water withholding period and (ii) a PreStress (PS) treatment, consisting of 1 week of complete water withholding followed by 1 week of growth under well-watered conditions and then the second water withholding period. The second water withholding period coincided with the only water withholding period of the NPS-treated plants. For experiments with Tienshan and Kopu, moisture content of the media (soil) was measured daily using a Time Domain Reflectometer (TDR; Trase Soil Moisture Measuring System, Soilmoisture Equipment Corp., Santa Barbara, CA, United States) with 15 cm soil probes, during the only (NPS) or second (PS) water withholding period. For experiments with Huia and derived RNAi lines, the moisture content of vermiculite and perlite mixture was determined using the gravimetric method (Robinson, 1974) and was expressed as a percentage using the formula 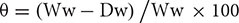; where, Ww: is the wet weight of vermiculite:perlite mixture at the time of sampling; Dw: is the dry weight of the vermiculite: perlite mixture.

### Nucleic Acid Isolation

For qRT-PCR, samples were collected daily until the moisture content dropped to approximately 9–10% for the soil grown plants Tienshan and Kopu and 32% for both the PS and NPS treatments of vermiculite:perlite grown Huia. Total RNA was extracted from different samples using the Hot Borate method (Hunter and Reid, 2001; Moser et al., 2004). The RNA/DNA concentration was determined by measuring the absorbance at 260 nm (A_260_) using a NanoDrop ND-1000 spectrophotometer V3.6 (Thermo Scientific, United States). Genomic DNA-free RNA samples were prepared according to the manufacturer’s instructions using an RNase-free recombinant DNase treatment (Roche Applied Sciences, Roche Diagnostics GmbH, Mannheim, Germany). Total RNA (2–10 μg) was mixed with 5 μL of 10× incubation buffer and 1 μL of DNase I (10 U), 1 μL of Protector RNase Inhibitor (10 U) and water to a final volume of 48.4 μL and incubated at 37°C for 20 min. The reaction was stopped by the addition of 1.6 μL of 0.25 M EDTA pH 8.0 (to make a final concentration of 8 mM) and was heated at 75°C for 10 min.

### Synthesis of cDNA and Quantitative Reverse-Transcription PCR (qRT-PCR)

The Transcriptor First Strand cDNA synthesis kit (Roche Applied Sciences) was used to synthesize single strand DNA according to the manufacturer’s instructions. In 0.2 mL capacity tubes, 1 μg of total RNA was combined with Oligo (dT)_15_ primer and the volume was adjusted with water to 13 μL. The mixture was denatured at 65°C for 10 min and subsequently placed on ice. Seven microliter of master reaction mixture (containing 5X transcriptor RT reaction buffer, 20 U/μL protector RNase inhibitor, 1 mM dNTP-Mix and 10 U/μL transcriptor reverse transcriptase) was then added and cDNA synthesis was carried out at 55°C for 30 min. Heat inactivation of Transcriptor Reverse Transcriptase was then performed at 85°C for 5 min. For qRT-PCR analysis, gene specific primers were used as shown in Supplementary Table 1. Two reference genes namely *Tr-*β*-actin* and *Tr-GAPDH* were used for qRT-PCR. The standard curve method was used to determine the efficiency of all the primer sets (Ruijter et al., 2009). The cDNA samples were diluted 20-fold using sterile water and qRT-PCR was performed using LightCycler^®^ 480 Real-Time PCR (Roche Applied Sciences). SYBR green I was used to monitor efficient DNA synthesis with three technical replicates for each 20-fold diluted cDNA sample. Total volume of each reaction was 10 μL consisting of 5 μL of 2 X LightCycler^®^ 480 SYBR Green I Master Mix (Roche Applied Sciences), 2.5 μL of 20-fold diluted cDNA and 0.5 μL of 10 μM forward and reverse primers and 1.5 μL of sterile water. PCR was performed using 96 well plates and the program was: 95°C for 5 min (95°C 10 s, 60°C 10 s, 72°C 10 s) × 40 cycles, 95°C melt and fluorescence measurements at 72°C for each cycle and continuously during final melting.

### Measurement of Proline Concentration

The leaf proline content was measured according to the method of Magne and Larher (1992). Approximately 50–70 mg of powdered leaf tissue was suspended in 1.2 mL of 3% (w/v) sulfosalicylic acid and the suspension was mixed vigorously using a vortex for approximately 30 s. The cellular debris was subsequently pelleted by centrifugation at 20,800 × *g* for 7 min at 4°C. Five hundred microliter of supernatant was transferred to a glass tube and 500 μL of water was added to a final volume of 1 mL. Two milliliter of extraction reagent, containing Ninhydrin (1% w/v) in 60% (v/v) glacial acetic acid, was then added to the tubes. The mixture was incubated in a water bath at 98°C for 1 h, after which the reaction was stopped by cooling the tubes on ice. To extract proline, 3 mL of toluene was added and the tubes were vortexed for 30 s and were then allowed to stand for 5 min to separate the toluene phase (containing proline) from the rest of the supernatant. The upper phase was then transferred to a 3 mL glass cuvette and the absorbance was measured at 518 nm against a toluene blank.

### Statistical Analysis

All the statistical analyses presented in this study were performed by SPSS 11.5 for Windows. Significance was tested using student’s *t*-test and *P* < 0.05 was considered as significant. For the qRT-PCR analysis for Tienshan and Kopu, pooled tissue from three independent plants, grown in individual pots was considered as a single biological replication. For wild type Huia cultivar plants, pooled tissue from four stolons of a single plant was considered as a biological replication. For the RNAi lines, pooled tissue from three genetically independent lines was considered as a biological replication. For each biological replication, qRT-PCR was repeated two or three times depending on the number of total samples for each experiment to minimize run to run and plate to plate variation. Relative transcript abundance was determined using the formula by Pfaffl (2001) and Pfaffl et al. (2004). For proline accumulation for the RNAi lines, pooled tissues from four genetically independent lines were used as a biological replication.

## Results

### Transcription of Tr-KPI Genes Is Inducible by Water Deficiency

To measure the effect of water limitation on the transcription of *Tr-KPIs*, we used white clover ecotype Tienshan, and two cultivars, Kopu and Huia, with different degrees of water requirements (Hofmann et al., 2003). Two treatments were applied to the plants: the first group designated as the NPS treatment directly received water deficit treatment. In the second group, designated as the PS treatment, the plants were subjected to a period of water deficiency followed by rehydration before exposure to the same water deficit period as the NPS treated plants.

**FIGURE 1:**
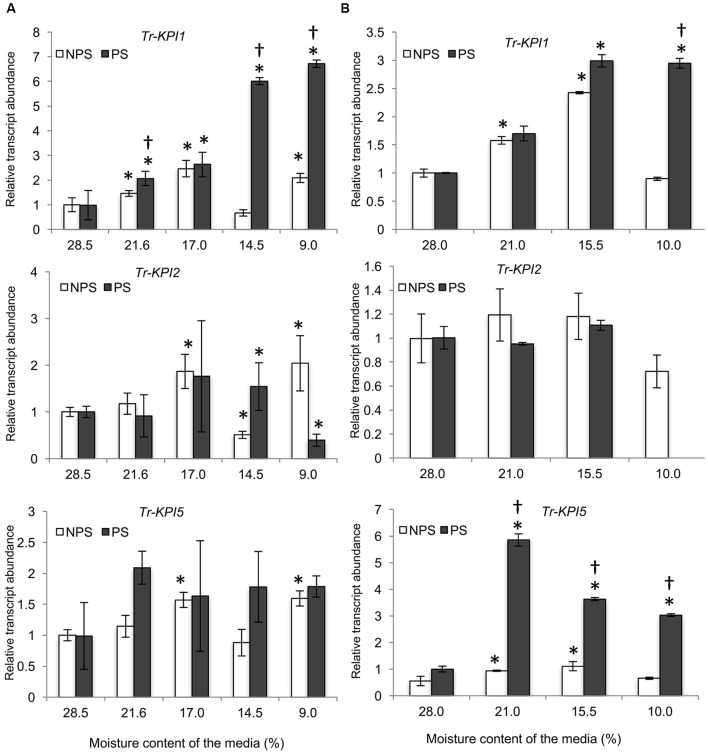
*Tr-KPI* transcript abundance is responsive to water deficit treatment in white clover. Transcription of *Tr-KPI1*, *Tr-KPI2*, and *Tr-KPI5* in the first fully expanded leaf (FFE) of white clover ecotype Tienshan **(A)** and cultivar Kopu **(B)**, as indicated, under NPS and PS treatments. Relative transcript abundance was determined by qRT-PCR using two biological replicates, with pooled tissues collected from at least three individual plants comprising a biological replicate, and was normalized using two internal reference genes, *Tr-*β*-actin* and *Tr-GAPDH*. The moisture content represent the mean value of the biological replicates and each data point represents mean value ± SE, of the biological replicates. Statistical analysis was performed using Student’s *t*-test. ^∗^Indicate statistically significant (*P* < 0.05) differential expression in comparison with initial moisture content for NPS and PS treatment, respectively, and ‘^†^’ indicates statistically significant (*P* < 0.05) expression in the PS treatment in comparison with the NPS treatments using Student’s *t*-test.

First, *Tr-KPI* gene expression was measured in response to the NPS and PS treatments in soil grown Tienshan and Kopu plants (**Figures 1A,B**): both water limitation conditions induced *Tr-KPI1* gene expression, but the transcript levels increased more in PS-treated plants. Transcript levels of *Tr-KPI2* did not vary much in Tienshan and Kopu grown under both NPS and PS conditions (**Figures 1A,B**). However, the expression of *Tr-KPI5* was only highly induced (sixfold) in the more drought sensitive Kopu cultivar in response to the PS treatment (**Figure 1B**).

**FIGURE 2:**
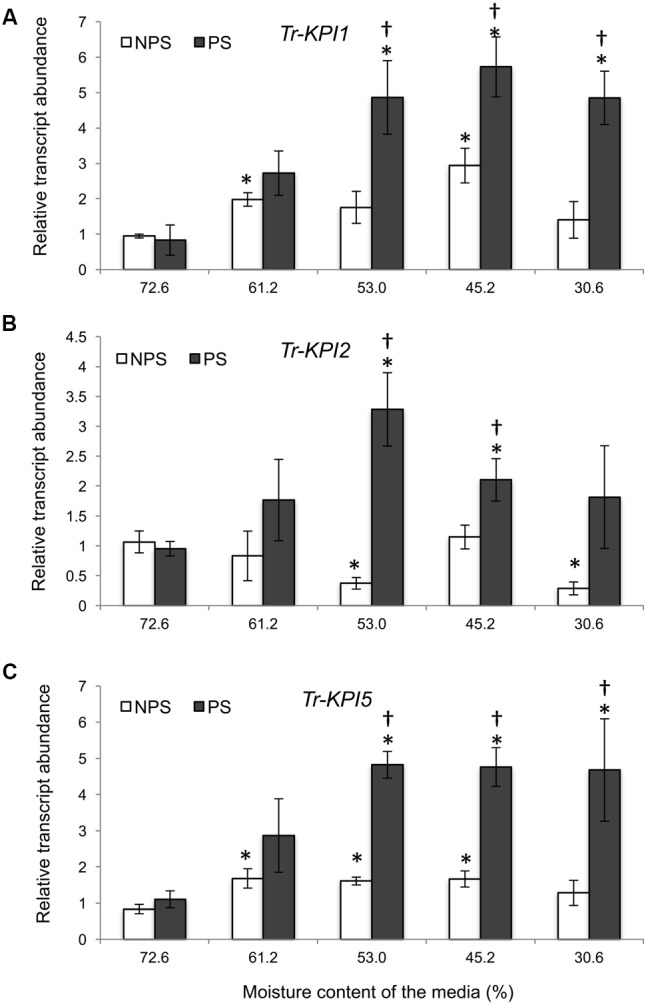
*Tr-KPI* transcript abundance is responsive to water deficit treatment in white clover cultivar ‘Huia.’ Relative transcript abundance of *Tr-KPI1* **(A)**, *Tr-KPI2* **(B)**, and *Tr-KPI5* **(C)** in the first fully expanded leaf (FFE) of white clover, as indicated, under NPS and PS treatments. Relative transcript abundance was determined by qRT-PCR using at least three biological replicates, with pooled tissues collected from at least three stolons comprising a biological replicate, and was normalized using two internal reference genes, *Tr-*β*-actin* and *Tr-GAPDH*. The moisture content represent the mean value of the biological replicates and each data point represents mean value ± SE, of the biological replicates. Statistical analysis was performed using Student’s *t*-test. ^∗^Indicate statistically significant (*P* < 0.05) differential expression with the initial moisture content of the NPS or PS treatment, respectively. ^†^Indicates statistically significant (*P* < 0.05) differential expression in the PS in comparison with the NPS treatments using Student’s *t*-test.

Next, Huia cultivar plants were grown on a defined vermiculite:perlite medium to allow isolation of RNA from root tissue. Here, *Tr-KPI1* expression started to increase significantly from 53% moisture content compared with the initial moisture content and with the NPS treatment (**Figure 2A**). However, *Tr-KPI2* expression increased significantly at 53 and 45.2%, while the expression of *Tr-KPI5* was fivefold induced in the PS treatment (**Figures 2B,C**). Furthermore, although the PS condition induced expression of the three *Tr-KPI* genes in the leaf tissue of Huia, in the root tissue only *Tr-KPI5* was induced, where a significant increase at 53% and an increasing trend at 45.2 and 30.6% (*P*-value 0.08 and 0.06, respectively) was observed when compared to the NPS treatment (Supplementary Figure 1D). These qRT-PCR results showed that expression of members of the *Tr-KPI* gene family, particularly *Tr-KPI1* and *Tr-KPI5*, increased during water limitation and the increase in transcript levels was stronger in response to the PS treatment.

### Water Deficiency Induces Physiological and Transcriptional Changes in White Clover

**FIGURE 3:**
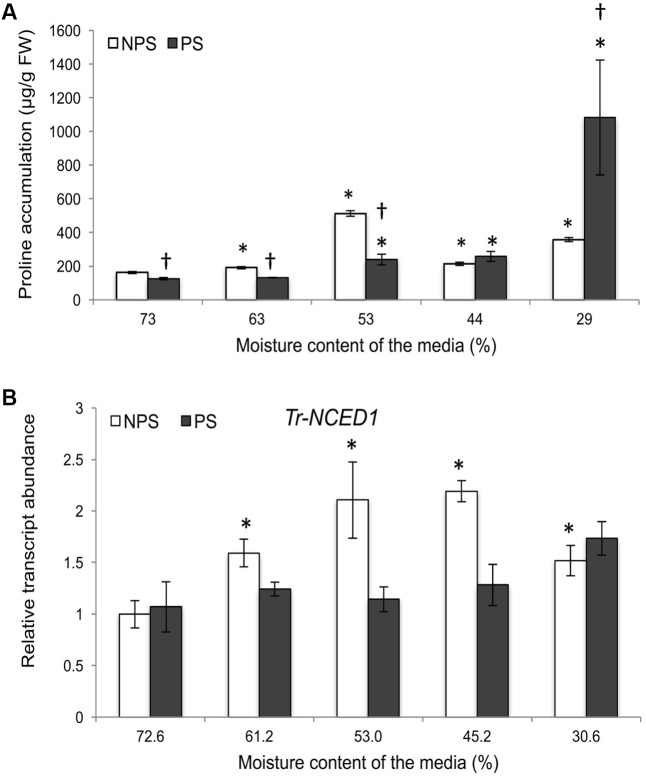
Water deficit treatments in white clover induce *Tr-NCED1* transcript upregulation and proline accumulation. Accumulation of L-Proline in wild type plant under PS and NPS treatments **(A)** and transcription of *Tr-NCED1* **(B)**, as indicated, in the first fully expanded (FFE) leaves in response to NPS and PS treatments against the moisture content of the media, as indicated. For L-proline accumulation, each value represents the mean from two biological replications each consisting of pooled tissues from four stolons. For *Tr-NCED1*, relative transcript abundance was determined by qRT-PCR using at least three biological replicates, with pooled tissues collected from at least three stolons comprising a biological replicate, and was normalized using two internal reference genes, *Tr-*β*-actin* and *Tr-GAPDH*. The moisture content represent the mean value of the biological replicates and each data point represents mean value ± SE, of the biological replicates. Statistical analysis was performed using Student’s *t*-test. ^∗^Indicate statistically significant (*P* < 0.05) differential expression in comparison with initial moisture content for NPS and PS treatment, respectively, and ‘^†^’ indicates statistically significant (*P* < 0.05) expression in the PS treatment in comparison with the NPS treatments using Student’s *t*-test.

As gene expression of three *Tr-KPIs* was induced in Huia, we used this variety for further study to determine additional responses of water limitation in NPS and PS treatments. Proline accumulation has been reported to increase under different environmental cues and it has been associated with metabolic changes during water deficit (Singh et al., 1972, 2000; Verbruggen and Hermans, 2008). Therefore, we measured the accumulaton of this amino acid in leaf tissue of plants grown under NPS and PS treatments. It was found that the NPS treatment resulted in significantly increased proline levels from 63% moisture content, while the PS treatment increased proline content from 53% moisture content. Moreover, the NPS treatment caused more proline accumulation than the PS treatment up to 44% moisture content (**Figure 3A**).

Further, we studied the expression of key genes in the ABA and Et biosynthesis pathway during the NPS and PS treatment as markers for stress imposed to the plants. The expression of *9-cis-epoxycarotenoid dioxygenase 1* (*NCED1*) has been reported to correlate with the accumulation of ABA upon water limitation (Qin and Zeevaart, 1999; Deluc et al., 2009; Speirs et al., 2013) and here we studied transcript levels of *Tr-NCED1*. Increased *Tr-NCED1* levels were observed only in the NPS treatment where it was significantly higher from 61.2 to 30.6% moisture content (**Figure 3B**). We also studied the genes encoding Et biosynthesis enzymes 1-aminocyclopropane-1-carboxylate (ACC) synthase (ACS) and ACC oxidase (ACO) (Supplementary Figure 2) as Et biosynthesis has been reported to increase upon water limitation (Apelbaum and Yang, 1981; Arraes et al., 2015). In the NPS treatment, a marginal significant increase was observed for *Tr-ACS1* at 45.2% and for *Tr-ACO1* at 30.6% moisture content (Supplementary Figure 2A). In the PS treatment, a decreasing trend was observed for *Tr-ACS1* and *Tr-ACO2* while *Tr-ACO1* displayed a significant increase at 30.6% moisture content (Supplementary Figure 2B). The results showed that NPS treatment induced early proline accumulation and the transcription of *Tr-NCED1* but affected Et biosynthesis genes only at lower moisture content levels. The results also suggest that a previous stress (PS treatment) prepared the plants for future stress, resulting in a lower proline accumulation without any significant up-regulation of ABA and limited up-regulation of Et biosynthesis genes (*Tr-ACO1*) during a subsequent drought period.

### Knock-Down of Tr-KPI Genes Result in Increased Proline Accumulation

To ascertain the function of *Tr-KPIs* in water limiting conditions, RNAi lines for four *Tr-KPI* genes in the cultivar Huia were generated. These lines were called *35S::tr-kpi1*, *35S::tr-kpi2 35S::tr-kpi4* and *35S::tr-kpi5* and are described in Islam et al. (2015a). For each construct, four independent lines were selected in which the targeted *Tr-KPI* was most effectively knocked-down (Supplementary Figure 3; Islam et al., 2015a). Although the RNAi constructs were designed to specifically knock down the targeted genes, untargeted *Tr-KPI* genes were also affected: in the leaf tissue of the *35S::tr-kpi1* knock-down lines, the expression of *Tr-KPI1* was reduced to 7%, *Tr-KPI2* to 9% and *Tr-KPI5* to 40%. In *35S::tr-kpi2* lines the expression of *Tr-KPI1* was reduced to 65%; *Tr-KPI2* to 2% and *Tr-KPI5* by 76 %. Finally, in *35S::tr-kpi5* lines, the expression of *Tr-KPI1* was reduced to 29%, *Tr-KPI2* to 18% and *Tr-KPI5* to 20% (Supplementary Figure 3; data was taken from Islam et al., 2015a). Nevertheless, the expression of *Tr-KPI1* and *Tr-KPI2* was lowest in their respective *35S::tr-kpi1* and *35S::tr-kpi2* lines while, in *35S::tr-kpi5* lines, expression of *Tr-KPI1*, *Tr-KPI2* and *Tr-KPI5* genes decreased to <30% of untransformed control lines.

**FIGURE 4:**
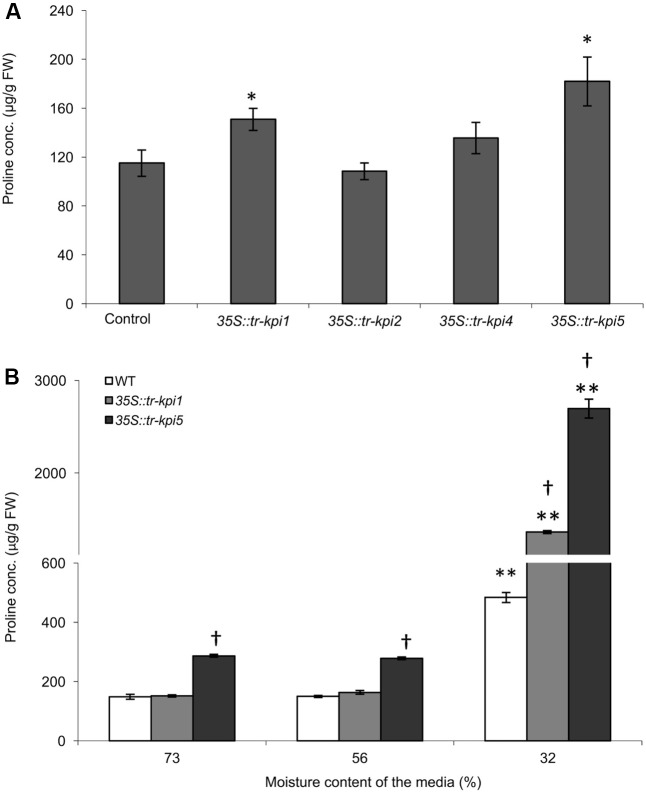
Accumulation of proline in *Tr-KPI* RNAi lines under well-watered conditions and during PS treatment. **(A)** Accumulation of proline in the RNAi lines. Each value represents the mean of four genetically independent lines (Values of individual lines shown in Supplementary Figure 4) (*n* = 4 for control, *35S::tr-kpi1*, *35S::tr-kpi2* and *35S::tr-kpi5*; *n* = 3 for *35S::tr-kpi4*). ^∗^Indicate statistically significant values in comparison with the control group (*P* < 0.05). **(B)** Proline accumulation under the PS treatment. Each value represents the mean ± SE, of three biological replications where each biological replication includes pooled tissue from four genetically independent lines. ^∗∗^Indicates statistically significant (*P* < 0.01) difference against the initial moisture content of the media. ^†^Indicates statistically significant (*P* < 0.01) difference in the PS treatment in comparison with the control group using Student’s *t*-test.

The selected *Tr-KPI* knock-down lines and wild type control plants were grown under well-watered and PS water limiting conditions and we did not observe any obvious visible differences between the wild type and RNAi lines (data not shown). To determine the effect of *Tr-KPI* knock-down on proline levels, we first measured proline content in the leaf tissue of these RNAi lines grown in well-watered conditions (**Figure 4A** and Supplementary Figure 4). We observed that *35S::tr-kpi1* and *35S::tr-kpi5* plants displayed significantly increased proline levels, when grown in well-watered conditions, whereas *35S::tr-kpi2* and *35S::tr-kpi4* (a root specific *KPI* gene) lines did not show any change compared to control plants (**Figure 4A** and Supplementary Figure 4). We also measured proline accumulation during water limitation in the PS treatment for *35S::tr-kpi1* and *35S::tr-kpi5* plants. In the PS time course study, the initial proline content was significantly higher in *35S::tr-kpi5* lines when compared to the wild type plants. Nevertheless, both *35S::tr-kpi1* and *35S::tr-kpi5* plants displayed a significantly higher proline content compared to the wild type plants at 32% moisture content (**Figure 4B**). These result show that the accumulation of proline in *35S::tr-kpi1* and *35S::tr-kpi5* lines is not a result of a water limiting condition *per se* but suggests an accentuated response in water limiting conditions.

### Knock-Down of Tr-KPI Genes Result in Increased Transcription of ET Biosynthesis Genes

**FIGURE 5:**
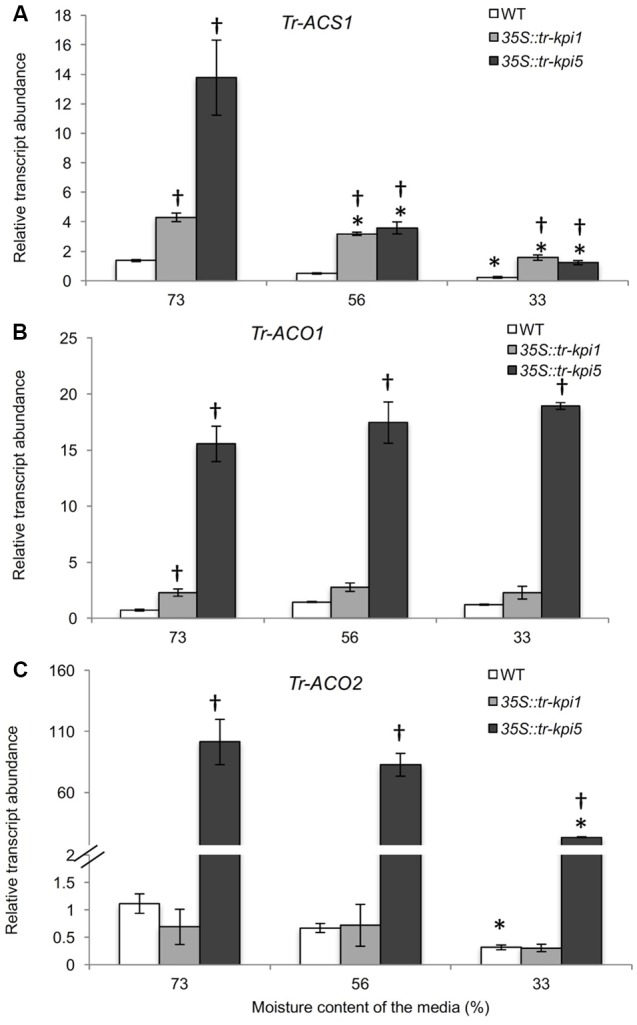
Changes in the transcript abundance of ET biosynthesis genes in the RNAi lines. Expression of the genes encoding ACC- synthase (*Tr-ACS1*) **(A)** and ACC-oxidase1(*Tr-ACO1*) **(B)** and ACC-oxidase 2 (*Tr-ACO2*) **(C)** in the first fully expanded leaf (FFE) of white clover RNAi lines in response to PS treatment against the moisture content of the media, as indicated. Relative transcript abundance was determined by qRT-PCR using two biological replicates, with pooled tissues collected from three genetically independent lines comprising a biological replicate, and was normalized using two internal reference genes, *Tr-*β*-actin* and *Tr-GAPDH*. Each data point represents mean value ± SE of the biological replicates (*n* = 2). Statistical analysis was performed using Student’s *t*-test. ^∗^Indicates statistically significant (*P* < 0.05) differential expression in comparison with initial moisture content. ^†^Indicates statistically significant (*P* < 0.05) expression in the PS treatment in comparison with control group using Student’s *t*-test.

The accumulation of increased proline in the RNAi lines grown in well-watered and PS treatment suggested increased stress. Therefore, genes encoding enzymes involved in Et biosynthesis were examined. Both *35S::tr-kpi1* and *35S*::*tr-kpi5* plants displayed significantly increased transcript abundance of *Tr-ACS1* and *Tr-ACO1* genes compared to the control plants (**Figures 5A,B**). The expression of *Tr-ACS1* decreased gradually with the decrease in moisture content, while there was no change in the transcript levels of *Tr-ACO1* compared with the initial moisture content in both RNAi lines. In *35S*::*tr-kpi5* lines, the transcript abundance of *Tr-ACO2* also remained significantly higher up to 33% moisture content as compared to the initial moisture content and to the control plants (**Figure 5C**). Therefore, *35S::tr-kpi5* lines responded more strongly than *35S::tr-kpi1* lines to water deficiency. The results suggest that the RNAi lines experience constitutive stress even in the absence of apparent adverse environmental conditions and that water deficiency enhanced the stress response.

## Discussion

Kunitz proteinase inhibitors play vital roles in plant development and defense (Ryan, 1990; Mosolov and Valueva, 2011; Boex-Fontvieille et al., 2015; Islam et al., 2015a,b). We have previously suggested that specific white clover KPIs may exert their functions by maintaining cellular homeostasis and here we hypothesized that altered homeostasis may affect abiotic stress responses as well and that KPIs may function during drought stress. Therefore, we examined the function of *KPI* expression on white clover plants that were subjected to two distinct water with-holding regimes. In the first NPS treatment, plants were subjected to a single water deficit period. In the second, PS treatment, plants were exposed to water deficit for a week followed by rehydration before being exposed to the same water deficit period as the NPS-treated plants. The PS treatment was performed with the aim to induce stress priming, which has been shown to result in a response that is earlier and stronger upon a successive biotic or abiotic stress treatment (Pastor et al., 2013; Borges et al., 2014). Over the course of the final or only water withholding treatment, several responses to water stress were measured in the plants: expression of the gene encoding the rate-determining enzyme in the ABA biosynthesis pathway, *NCED1* (*Tr-NCED1*) was measured. In addition, expression of *ACC synthase* (*Tr-ACS1*) and *ACC oxidase* (*Tr-ACO1* and *Tr-ACO2*), encoding proteins involved in ET biosynthesis, was quantified and finally, proline accumulation was determined.

In NPS treated plants, the transcript levels of *Tr-NCED1* significantly increased, suggesting that ABA biosynthesis was up-regulated (**Figure 3**). An increase in the transcript levels of Et biosynthesis gene *Tr-ACS1* and an early significant increase in proline accumulation was also observed (**Figure 3** and Supplementary Figure 2). The up regulation of ABA and Et biosynthesis genes, together with proline accumulation during the NPS treatment suggests a stress response to water deficiency. In contrast, the PS treatment did not result in a change in *Tr-NCED1* transcript levels and a down-regulation of *Tr-ACO2* was observed (**Figure 3** and Supplementary Figure 2). Further, delayed as compared to NPS-treated plants, but significant accumulation of proline was detected in the PS treated leaf tissue (**Figure 3**). Therefore, stable *Tr-NCED1* expression in PS-treated plants suggests unchanged ABA biosynthesis and/or the involvement of other, as yet unidentified, members of the *NCED* gene family in ABA biosynthesis. Thus, the overall response in NPS treatment suggests a possible involvement of ABA-dependent pathway; whereas, the findings of the PS treatment are consistent with a greater drought tolerance and/or the involvement of an ABA-independent pathway to govern the drought response (Shinozaki and Yamaguchi-Shinozaki, 1997; Bray, 2002; Zhu, 2002; Shinozaki et al., 2003; Verslues and Bray, 2006; Planchet et al., 2011). Consequently, the findings of the PS treatment suggest a ‘stress imprint’ from a previous water deficiency that prevented the plants from having to bear the costs associated with triggering early proline accumulation and an ABA response (Conrath et al., 2006; Bruce et al., 2007; Walter et al., 2011).

As marked differences were observed in the NPS and PS treatment, we hypothesized that *Tr-KPI* gene expression would respond differently to NPS and PS treatments. In this study, we found that the expression of selected *Tr-KPI* genes was induced significantly in the NPS treatment in aboveground, but not in root tissues (**Figures 1**, **2** and Supplementary Figure 1). Our results are similar to findings of Kang et al. (2001) who reported that KPI-like proteins are induced by water deficiency and is broadly in agreement with data from Downing et al. (1992), who detected tissue specific expression of *KPIs* in *Brassica napus* leaves, but not in roots or seed. Nevertheless, we cannot exclude the possibility that other members of *Tr-KPI* gene family may respond to water deficiency in root tissue. In summary, the findings of the NPS treatment are in support of a role for *Tr-KPIs* in water deficiency.

Since the PS treatment resulted in a reduced stress response, we expected decreased induction of *Tr-KPIs* upon the second drought stress. Surprisingly we found that in the PS treatment the levels of *Tr-KPIs* transcripts were significantly induced, even in comparison with the NPS treatment. Of the three genes studied, *Tr-KPI5* was most highly responsive in the more drought-sensitive varieties, signifying a possible function of this gene in water deficiency. These results are consistent with the idea that increased *Tr-KPI* expression may actually function as part of a mechanism that allow the plant to better cope with the drought stress, especially the second drought period of the PS treatment. Therefore, to examine the functional implication of transcriptional regulation of *Tr-KPIs*, especially *Tr-KPI5* more directly in water deficiency, RNAi knock-down plants for the studied genes were developed in the Huia background.

We have shown earlier that the leaves from the knocked-down plants displayed some stress-associated cellular signatures including an increase in H_2_O_2_ levels and a concurrent increase in SOD activity (Islam et al., 2015a). In this study, we observed that the *35S::tr-kpi1* and *35S::tr-kpi5* RNAi lines accumulated more proline than the wild type when grown in well-watered conditions. The higher proline accumulation may have a direct or indirect function in scavenging reactive oxygen species (ROS) (Alia et al., 2001; Liang et al., 2013; Signorelli et al., 2014; Zhang and Becker, 2015; Zhang et al., 2015). We found previously that *Tr-NCED1* transcript levels remain unchanged in *35S::tr-kpi1* and *35S::tr-kpi5* lines compared to the controls during well-watered growth conditions (Islam et al., 2015a), suggesting that increased proline content in these plants are not related to the expression of *Tr-NCED1*. Because the *35S::tr-kpi2* lines did not accumulate higher proline levels, the results eliminate the possibility that the increased proline levels in the *35S::tr-kpi1* and *35S::tr-kpi5* lines is the result of *Tr-KPI2* knock-down. Nevertheless, the results suggest that the *Tr-KPI1* and *Tr-KPI5* knock-down plants experience constitutive stress. We employed a PS treatment to investigate the effect of *Tr-KPI* knock-down on the response to water deficiency. The *35S::tr-kpi5* RNAi lines accumulated significantly more proline in the PS treatment than the wild type, while higher proline levels in *35S::tr-kpi1* lines were only found at the lowest moisture level. Additionally, an increased transcription of Et biosynthesis genes were observed in the leaves of the RNAi lines, particularly of the *Tr-KPI5* knocked-down lines (**Figure 5**). The induction of Et biosynthesis genes did not coincide with visible Et-induced senescence as we did not observe any visual yellowing of the treated plants (Oh et al., 1997; Arraes et al., 2015; data not shown). Therefore, the result suggests that upregulated transcription of *Tr-KPI5* may function to better cope with drought stress in the drought-susceptible varieties. Hence, we propose that the increased proline accumulation in *35S::tr-kpi1* background resulted from the transcriptional suppression of *Tr-KPI5* (Supplementary Figure 3). As the knock-down of *Tr-KPIs* correspond with increased proline content in the RNAi plants, the results also reveal the correlation of up-regulated expression of *Tr-KPIs* and reduced proline content in stress adapted PS-treated plants.

Previously, we reported that the transcription of *Tr-KPI1* is highly responsive to biotic stress such as mechanical wounding and insect herbivory (Islam et al., 2015a). The knocked-down *35S::tr-kpi1* lines displayed the most marked reduction in herbivory feeding as shown by the retarded larval development compared to wild type plants and *35S::tr-kpi2* and *35S::tr-kpi5* lines. Instead, *Tr-KPI2* plays a central role in the development of white clover as the *35S::tr-kpi2* knocked-down lines produced shorter stolons and displayed reduced branching. The seven other members of the *Tr-KPI* gene family could have evolved to acquire different or overlapping functions in plant development and metabolism (Islam et al., 2015a). Therefore, we propose that members of the *Tr-KPI* multi-gene family have preferential functions in regulating cellular homeostasis and stress resistance such that *Tr-KPI1* may function mainly as a result of biotic stress; *Tr-KPI2* during development and *Tr-KPI5* in response to abiotic stress.

The current study highlighted that specific *Tr-KPI* genes are involved in water stress response and future studies should focus on the elucidation of the underlying molecular mechanism. Drought stress can cause a marked increase in cellular proteolytic activity in susceptible plants (Degenkolbe et al., 2009; Simova-Stoilova et al., 2010; Mosolov and Valueva, 2011) and therefore, the wider *Tr-KPI* gene family has the potential to regulate proteolysis during water deficiency. It is likely that *Tr-KPIs* are functionally diverse and target different *in planta* proteinases. Although, KPI proteins were found to have different *in vitro* targets, including proteinases (Ritonja et al., 1990; Terada et al., 1994; Valueva et al., 2000; Heibges et al., 2003b; Major and Constabel, 2008), there is little known about the *in planta* targets of constitutively expressed KPIs because of the presence of a large number of proteinase coding genes in plants. For example, the *Arabidopsis thaliana* genome encodes over 800 proteinases which exemplifies the difficulty to identify the *in planta* PI targets (van der Hoorn, 2008). We showed in a previous study that total trypsin inhibitory activity was not altered in any of the RNAi lines which suggests limited, if any, significant induction of other functional *Tr-KPI* proteins. Nevertheless, water deficiency can induce various classes of proteinases (Seki et al., 2002; Bartels and Sunkar, 2005; Kidric et al., 2014) and therefore, finding the proteinase targets of the Tr-KPI family deserves more attention.

In summary, from the expressional analysis and use of knocked-down *Tr-KPI* lines, we propose that particularly *Tr-KPI5* serves a central function under water limiting conditions in white clover. Therefore, these results are in support of our hypothesis that regulated expression of *Tr-KPIs* is of importance during water deficiency, possibly through alteration of cellular homeostasis. Our findings ascertain the involvement of the *Tr-KPI* gene family in water stress and add to existing evidence that the function of KPIs is not solely restricted to one of storage protein or in response to biotic stress. Thus, we propose that the regulation of expression of the *Tr-KPI* gene family is part of an intrinsic mechanism by which critical cell functions are controlled under water limiting conditions: if such control is disrupted then changes to cellular homeostasis occur and major stress-response changes ensue.

## Author Contributions

AI jointly conceived the study, acquired and interpreted the data, wrote the manuscript and assisted with manuscript revision. SL and AN acquired and analyzed data and commented on the manuscript. PD assisted with data interpretation, manuscript writing, commented, reviewed and submitted the manuscript. MM jointly conceived the study, interpreted the data, commented on the results. All the authors have read and approved the final version of the manuscript.

## Conflict of Interest Statement

The authors declare that the research was conducted in the absence of any commercial or financial relationships that could be construed as a potential conflict of interest.
