# Supplementary material for: Kunitz Proteinase Inhibitors Limit Water Stress Responses in White Clover (Trifolium repens L.) Plants
Source: Front Plant Sci. 2017 Oct 4;8:1683. doi: 10.3389/fpls.2017.01683 (PMC5632647; doi:10.3389/fpls.2017.01683)
Supplement: Supplementary file 1 [file Data_Sheet_1.PDF]

Supplementary Material

Kunitz proteinase inhibitors limit hallmarks of water stress in white clover (*Trifolium repens* L.) plants

Afsana Islam, Susanna Leung, Aluh Nikmatullah, Paul P Dijkwel, Michael T McManus.

Corresponding Author:
Paul P Dijkwel; E-mail: p.dijkwel@massey.ac.nz

Supplementary Tables and Figures

Supplementary Table 1 Sequences of primers used for qRT-PCR.

**Gene**	**Sense**	**Antisense**
Tr-β-ACTIN	CGTATGAGCAAGGAGATCACTG	CATCTGCTGGAAGGTGCT
Tr-GAPDH	TCCAGTATTGAACGGTAAATTGAC	TCTGATTCCTCCTTGATAGCAG
Tr-KPI1	GGTAACGCCATCTTCCCAG	CTACGATCTTGTAGGACAGTAACC
Tr-KPI2	GGGAGATCTGGTAATGTGACAG	TCAAGGTATCAAACAACAGACTTAAT
Tr-KPI4	GATATTGGAAGGCATGATGATGAG	GTCTAACAAGTGATCAGCTAACCT
Tr-KPI5	CCATCCCACAAACCACCAC	GGTCCACCAATACCAACATAGC
Tr-ACS1	AGGTTTCGATCGAGATTTGA	CATCTGCTGGAAGGTGCT
Tr-ACO2	CTTGTAAAAGGTCTCCGAGCAC	GAGGAACATCTACCCATTTACCAT
Tr-ACO3	AGCATCATTCTACAACCCTGG	CAAACACAAATTTAGGATACACATTGG

Supplementary Figures

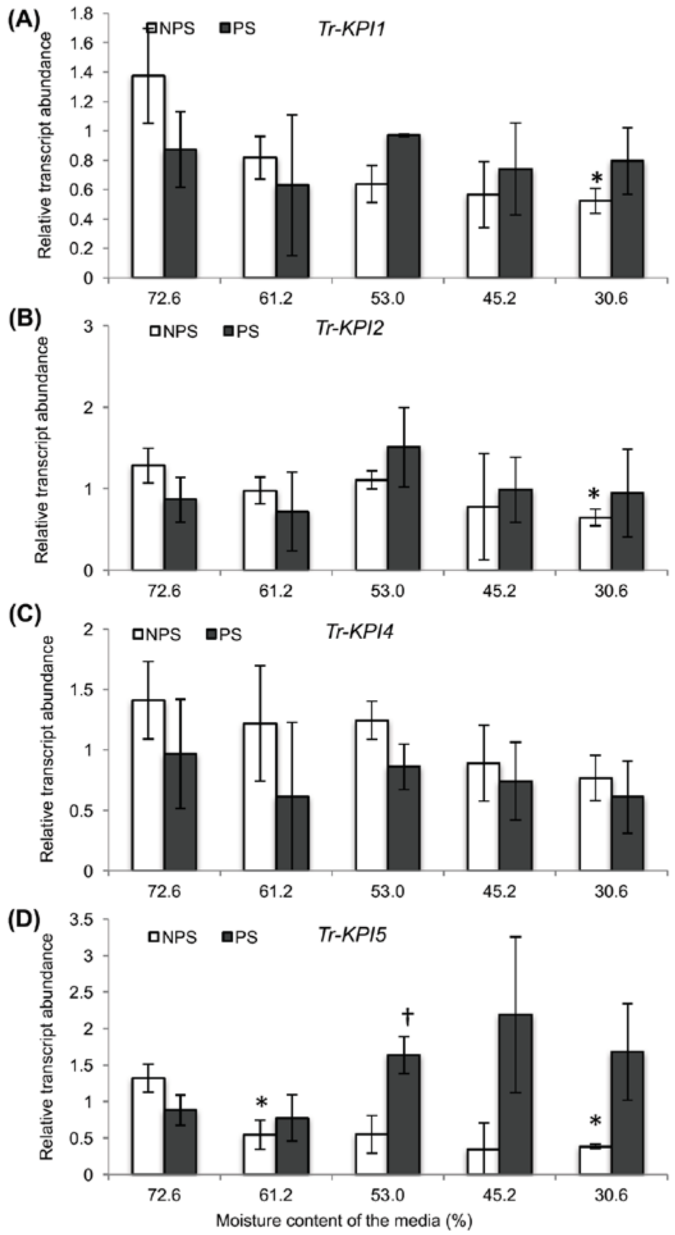

Supplementary Figure 1: *Tr-KPI* transcript abundance is responsive to water deficit treatment in white clover cultivar 'Huia'. Transcription of *Tr-KPI1* (A), *Tr-KPI2* (B), *Tr-KPI4* (C) and *Tr-KPI5* (D) in the root tissue of white clover, as indicated, in NPS and PS treatments. Relative transcript abundance was determined by qRT-PCR using at least three biological replicates, with pooled tissues collected from at least three stolons comprising a biological replicate, and was normalized using two internal reference genes, *Tr-β-actin* and *Tr-GAPDH*. Each data point represents mean value, ± SE, of the biological replicates. Statistical analysis was performed using Student's T-test. '*' indicates statistically significant ($P<0.05$) differential expression in comparison with initial moisture content for NPS and PS treatment respectively and '†' indicates statistically significant ($P<0.05$) expression in the PS treatment in comparison with the NPS treatments using Student's T-test.

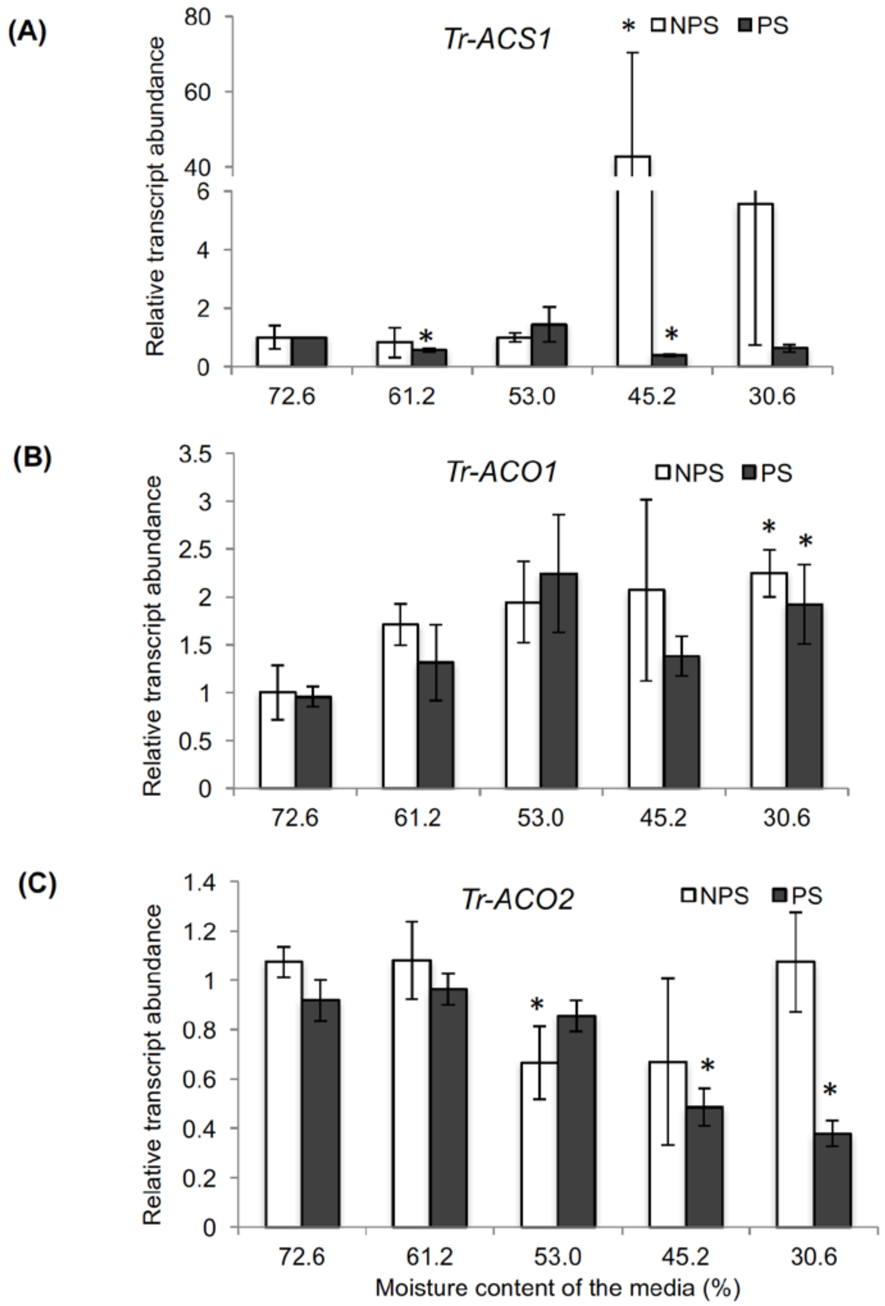

Supplementary Figure 2: Expression of AAC- synthase (*Tr-ACS1*) (A) and ACC-oxidase (*Tr-ACO1* and *Tr-ACO2*) (B and C) in the first fully expanded (FFE) leaf of white clover, as indicated, under NPS and PS treatments. Relative transcript abundance was determined by qRT-PCR using at least three biological replicates, with pooled tissues collected from at least three stolons comprising a biological replicate, and was normalized using two internal reference genes, *Tr-β-actin* and *Tr-GAPDH*. Each data point represents mean value, ± SE, of the biological replicates. Statistical analysis was performed using Student's T-test. '*' indicates statistically significant ($P<0.05$) differential expression in comparison with initial moisture content for NPS and PS treatment respectively and '†' indicates statistically significant ($P<0.05$) expression in the PS treatment in comparison with the NPS treatments using Student's T-test.

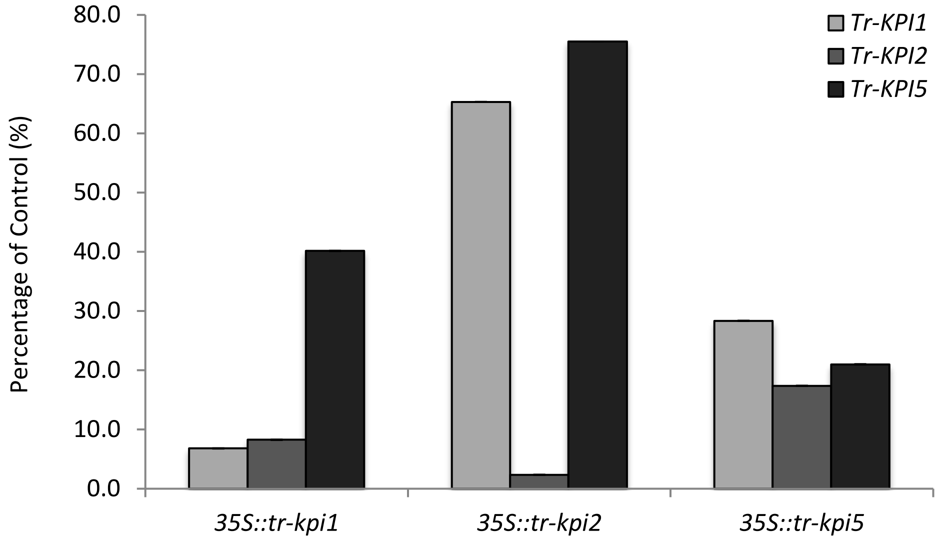

Supplementary Figure 3 Transcription of *Tr-KPI1*, *Tr-KPI2* and *Tr-KPI5* in the leaf tissue of *35S::tr-kpi1*, *35S::tr-kpi2* and *35S::tr-kpi5* RNAi plants, as indicated as percentage of control. Data is taken from Islam et al. (2015a). Each value represent the mean ± SE of four genetically independent knock-down lines as biological replicates (*n* = 4). Relative transcript abundance was determined by qRT-PCR and transcripiton was normalised against two internal reference genes *Tr-β-ACTIN* and *Tr-GAPDH*.

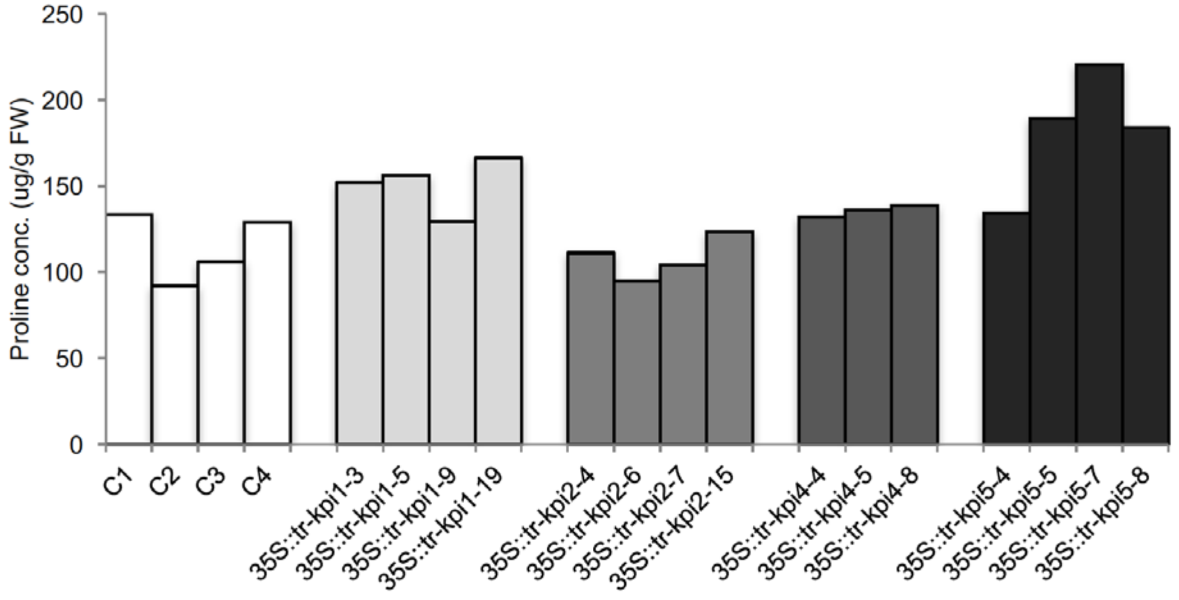

Supplementary Figure 4: L-proline accumulation in the first fully expanded (FFE) leaf of genetically independent T_0 lines of *35S::tr-kpi1*, *35S::tr-kpi2*, *35S::tr-kpi4* or *35S::tr-kpi5* or controls (C1, C2, C3 and C4), as indicated. Each value represent the mean of 15 pooled FFE leaves excised from sufficient stolons from vegetatively propagated plants for each line.
